# Tert-butylhydroquinone mitigates Carbon Tetrachloride induced Hepatic Injury in mice

**DOI:** 10.7150/ijms.45842

**Published:** 2020-07-29

**Authors:** Ruidong Li, Peng Zhang, Chengguo Li, Wenchang Yang, Yuping Yin, Kaixiong Tao

**Affiliations:** Department of Gastrointestinal Surgery, Union Hospital, Tongji Medical College, Huazhong University of Science and Technology, Wuhan, 430022, China.

**Keywords:** Tert-butylhydroquinone, liver injury, Nrf2, oxidative stress

## Abstract

Tert-butylhydroquinone (tBHQ) is an antioxidant compound that exhibits cytoprotective effect in many tissues under pathological condition. However, its role in carbon tetrachloride (CCL4) induced liver injury is still unclear. Here we established a carbon tetrachloride induced hepatic injury model in mice to determine whether tBHQ can mitigate CCL4 induced liver damage. In our study, we found tBHQ exhibited protective effects in CCL4 treated mice model. TBHQ markedly improved hepatic function and decreased hepatic histopathological damage *in vivo*. In addition, tBHQ reduced levels of pro-inflammatory cytokines in mice model. Moreover, tBHQ mitigated apoptosis of hepatocytes, oxidative stress and lipid peroxidation *in vivo* and *in vitro*. We also found the possible mechanism of protective effects of tBHQ was associated with activation of Nrf2/ heme oxygenase-1 (HO-1) pathway. In conclusion, our study revealed tBHQ can be a potential therapeutic drug in treatment of acute hepatic injury.

## Introduction

The liver is a pivotal organ that possesses important function in metabolism and oxidative response. Acute hepatic injury (ALI) leads to a lethal condition if the pathogenic factors are not removed. Hepatic damage is induced by many irritants including chemicals, viruses, infections and other hepatotoxic agents 1, 2. Despite the many mechanisms involved in hepatocyte damage oxidative injury caused by free radicals and reactive oxygen species (ROS) is leading pathogenesis in ALI 3. Carbon tetrachloride (CCL4) is widely used to induce ALI model in mice in large ranges of laboratory. Previous reports demonstrated that CCL4 is metabolized via p450 enzyme system, which produced trichloromethyl radical and trichloromethyl peroxy radical. These free radicals initiate lipid peroxidation and consequently result in ROS overproduction and hepatic damage 4.

Nuclear factor E2-related factor 2 (Nrf2) is named as it can bind erythroid-derived 2/activator protein 1 (NF-E2/AP1) repeat of the beta-globin gene 5. After activation by stimuli, it translocates into nucleus where it induces transcription of target genes related to xenobiotic detoxification, antioxidant system 6.Currently, it is extensively studied for regulating expression of a variety of antioxidant and detoxification genes 6. NAD(P)H: quinone oxidoreductase 1 (NQO1) is a pivotal endogenous antioxidant. Enhancing Nrf2 expression leads to increasing NQO1 level in mice 7. Therefore, Nrf2 is central regulator in expression of NQO1. Moreover, Nrf2 is involved in induction superoxide dismutase (SOD) to against ROS 8. SOD is antioxidant enzyme for scavenging oxygen free radicals, especially superoxide anion free radicals. Therefore, Nrf2 is an important antioxidative regulator which regulates function of many antioxidative proteins.

Tert-butylhydroquinone (tBHQ) is an antioxidant chemical that shows cytoprotective effect. *In vitro*, it protected against doxorubicin-induced cardiotoxicity by activating Nrf2 pathway, promoting Akt phosphorylation and suppression of oxidant-induced apoptosis 9. *In vivo*, treatment of tBHQ showed significant cytoprotective effect in different organs under pathological conditions associated or not with Nrf2 activation 10, 11. However, so far it is not clear whether tBHQ has protective effect in CCL4 induced liver injury. To investigate effect of tBHQ in CCL4 treated mice, we established CCL4 induced ALI model according to our previous report 12. Our data revealed that tBHQ showed protective effect in ALI model and reduced apoptosis and oxidative stress in hepatocytes. Furthermore, the activation of Nrf2 pathway was involved in this protective effect.

## Materials and Methods

### Animals

All adult male C57bl/6 mice aged 8-10 weeks were obtained from Beijing Huafukang Biotechnology Co., Ltd. (Beijing, China). All mice weights range from 20-25 g. The experiments were approved by the Animal Care and Use Committee of Tongji Medical College of Huazhong University of Science and Technology. All animals were maintained in standard environment and fed with laboratory food and water.

### Mouse experiment procedure

Hepatic damage was caused via injecting (i.p.) CCL4 with dosage of 0.1ml/kg (10ml/kg body weight, volume^CCL4^/volume^olive oil^=1:99). tBHQ was dissolved in pure dimethly sulfoxide (DMSO) then diluted in phosphate buffered saline (PBS) with a final concentration of 5 mg/ml and 1% DMSO in PBS. The injection (i.p.) of tBHQ was administrated into three times at intervals of 8 hours (0h, 8h and 16h). The dose of each injection was 16.7 mg/kg. Mice were divided into 4 groups (n=6): (1) Control group, same volume vehicle during entire procedure. (2) CCL4 group, CCL4 (0.1ml/kg (10 ml/kg body weight, volume^CCL4^/volume^olive oil^=1:99) was given once and then received same vehicle procedures as CCl4 + tBHQ group. (3) CCl4 + tBHQ group, mice received same dose of CCL4 as CCl4 group then were immediately treated with tBHQ (16.7 mk/kg/per times, three times, each 8h intervals). (4) tBHQ group, mice received tBHQ same dose and time points as CCL4+tBHQ group without CCL4 treatment. 24h after CCL4 treatment, animals were anesthetized with 60 mg/kg of 2% sodium pentobarbital then blood was collected. Mice were killed by cervical luxation. Liver was collected immediately and divided into two pieces. One half was fixed in paraformaldehyde for histopathologic analysis and the other was used for other analysis. All samples were collected 24 h after CCL4 treatment.

### Reagents

Tert-butylhydroquinone (purity: >97.0%) was purchased from Sigma Aldrich (St. Louis, MO, USA). CCl4 (purity>98%) was purchased from Acmec biochemical (Shanghai, China). The kits for measurement levels of alanine transaminase (ALT), reduced glutathione/oxidized glutathione (GSH/GSSG) level, aspartate transaminase (AST), malondialdehyde (MDA), and olive oil were purchased from the Nanjing Jiancheng Institute of Biotechnology (Nanjing, China). enzyme-linked immunosorbent assay (ELISA) kits for detection of mice interleukin-6(IL-6), interleukin-1β (IL-1β), interferon-γ (IFN-γ), monocyte chemotactic protein 1 (MCP-1) and Tumor necrosis factor-α (TNF-α) were purchased form Dakewe Bioengineering Co., Ltd. Fetal bovine serum and RPMI-1640 medium were obtained from Gibco Life Technologies (Carlsbad, CA, USA). Annexin V-FITC Apoptosis Detection Kit, Caspase-3 level detection kit and 2,7-Dichlorofluorescein diacetate (DCFH-DA) was purchased from Beyotime Biotechnology (Shanghai, China). Anti-heme oxygenase-1 (HO-1), anti-Nrf-2 and b-actin were obtained from Cell Signaling Technology (Danvers, Mass). TUNEL Apoptosis Assay Kit (ROCHE *In situ* Cell Death Detection Kit, TMR red) was purchased from Roche. All other chemicals were of the highest commercial grade.

### Histological examination of liver tissues

Hepatic tissues were inflated with 10ml H2O fixed with 4% paraformaldehyde, then embedded in paraffin. Sections were stained by hematoxylin-eosin (H&E) staining then images were captured by light microscope (Olympus IX71, Tokyo, Japan). Six fields were selected randomly and the necrosis area was independently evaluated by two pathologists according to morphologic criteria. Percentage of area was assessed by Image J.

### Measurement of hepatic enzyme

Serum was obtained via centrifugation of whole blood samples at speed of 800 g for 15 min. the level of ALT and AST were recorded by using ALT and AST assay kits according to manufacturer's instructions.

### TUNEL Assay

Briefly, liver paraffin-embedded sections were deparaffinized using ethanol. Then sections were incubated with TUNEL reaction mixture according to producer's instruction. Six random fields were selected randomly and number positive cells (positive cells/per fields) were calculated by Image J. Pictures were captures as described before.

### Evaluation of intracellular antioxidant level

Liver tissues were homogenized with ice-cold PBS and supernatants were collected. The GSH/GSSG activities and MDA were measured using respectively kits according to manufacturer's instruction.

### Cytokine quantification by ELISA

Mice serum samples were collected and activities of interleukin-6, interleukin-1β, interferon-γ, monocyte chemotactic protein 1 and Tumor necrosis factor-α were measured by ELISA kits according to manufacturer's instruction.

### Cell culture and treatment

Human hepatic carcinoma cell line (HepG2) was bought from TypicalCell Culture Collection Committee of the Chinese Academy of Sciences Library. The cell line was cultured in RPMI-1640 with 10% FBS. The exponential growth phase cells were grown in 6 well plates at 2 × 10^5^ cells/well overnight in 2ml culture medium. Then CCL4 group were treated with CCL4 (0.5% v/v). Tert-butylhydroquinone was dissolved in DMSO at concentration of 5 mM. Tert-butylhydroquinone in DMSO (5 mM, 2 ul) was added immediately after CCL4 treatment in CCL4+tBHQ group reaching final concentration at 5 uM. DMSO (2 ul) was used as vehicle and added in each group as a control. The cells were harvested for next analysis 24 h after CCL4 administration.

### ROS assay in HepG2 cells

ROS activities in cells were measured using DCFH-DA as a fluorescent probe previously described 12. Briefly, HepG2 cells were treated with final concentration of 10 µM DCFH-DA for 30 mins after CCL4 or/and tert-butylhydroquinone treatment. Then culture medium was removed and cells were washed with PBS for three times. The ROS levels of cells were recorded by FACSCanto II flow cytometer (BDBiosciences, SanJose, CA, USA). Data was analyzed using FCS express 3 (De Novo Software). The images were captured using fluorescence microscope (OlympusIX71, Tokyo, Japan).

### Apoptosis assay in HepG2 cells

Annexin V/propidium iodide staining was used to analyze apoptosis. In brief, cells were treated as describe above then harvested, washed with PBS and stained with apoptosis detection kit according to manufacturer's instruction. The data was visualized and analyzed by using FCS express 3 (De Novo Software).

### RNA interference

HepG2 cells were grown in 6 well plates and cultured for 24h reaching a confluence of 40%. Transfection was performed with Lipofectamine^®^ 2000 according to manufacturer's instruction. 12 h after transfection, cells were treated with CCL4 and tert-butylhydroquinone for another 24 h. Then cells were collected for next analysis.

### Western Blot

Cells after treatment were washed twice with ice cold PBS and lysed on ice for 20 min. the whole protein was extracted using a commercial kit (Beyotime, China). Supernatant protein concentrations were measured with BCA Protein Assay Kit (Beyotime, China). Cell lysates were separated in 10% poly acrylamide sodium dodecyl sulfate gels and then were transferred to PVDF membrane. Then PVDF was blocked in 5% non-fat milk for 1h at room temperature. Primary antibody against Nrf2 (1:1000), HO-1 (1:1000), b-actin (1:1000) was incubated with membrane overnight. Second antibody conjugated with horseradish peroxidase (HRP) was incubated with membranes for 1h next day. The proteins were visualized using commercial kit (Beyotime, China) and images were captured using UVP imaging system.

### Statistical analysis

Data were expressed as means ± SEM. Statistical analysis was performed using one-way analysis of variance (ANOVA) plus Tukey^,^s multiple comparison test. Statistical analyses were performed using Prism 8. *p*< 0.05 was considered significant.

## Results

### Tert-butylhydroquinone reduces CCL4 induced hepatic pathology

**Figure 1:**
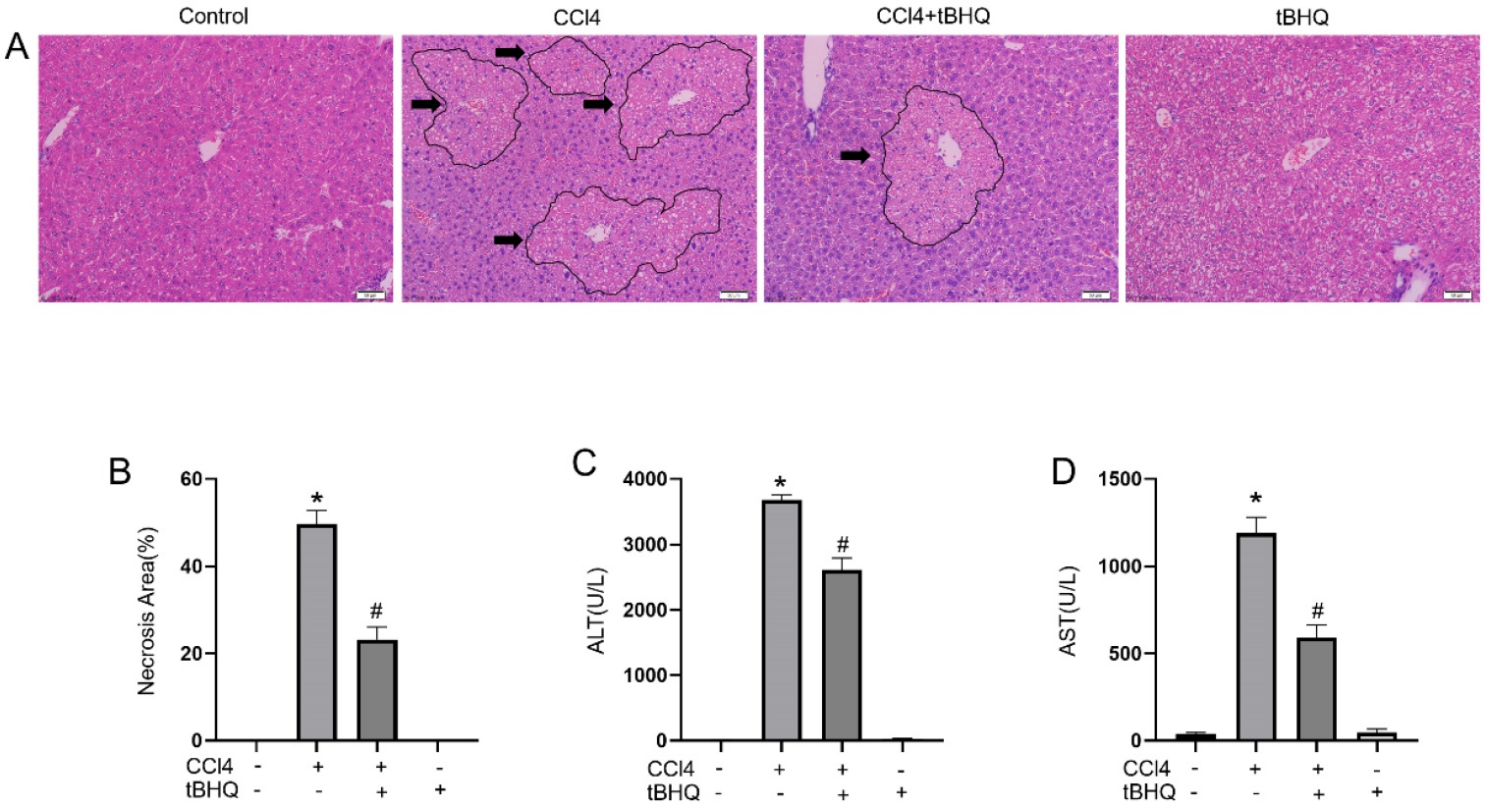
** Treatment of tBHQ mitigates CCL4 induced acute hepatic damage.** Representative micrographs from and necrotic areas in control mice, Vehicle+CCL4, tBHQ+CCL4 and single tBHQ according to hematoxyl in and eosin staining of hepatic sections. The arrows in images are labeled as necrotic area. CCL4 induced markedly necrosis while tBHQ decreased area of necrosis (**A**). The necrotic areas of each group were statistical analyzed (**B**). The serum activities of alanine aminotransferase (**C**) and aspartate aminotransferase (**D**) in CCL4 and/or tBHQ treated mice. Magnification 200x. Arrows imply area of liver necrosis. Data are expressed as mean ± SEM (n = 6). *p < 0.05 vs. control; #*p* < 0.05 vs. the CCL4 group.

We examined protective effect of tert-butylhydroquinone in CCL4 induced liver injury. As shown in Fig. 1A, H&E staining of liver sections showed CCL4 can cause markedly hepatic massive cell necrosis and loss of hepatocyte architecture. However, administration of tert-butylhydroquinone significant reduced histopathological damage and necrosis. In addition, tert-butylhydroquinone alone displayed no histopathological damage.

### Tert-butylhydroquinone protects liver function

The levels of ALT and AST are considered as important markers to evaluated hepatic function. We found CCL4 can significantly increase activities of ALT and AST compared to control group. Administration of CCL4 treated mice with tert-butylhydroquinone markedly reduced levels of AST and ALT (Fig. 1C and 1D). And mice received tert-butylhydroquinone alone showed no impact on liver function.

### Tert-butylhydroquinone attenuates hepatocytes apoptosis and reduces oxidative stress *in vivo*

**Figure 2:**
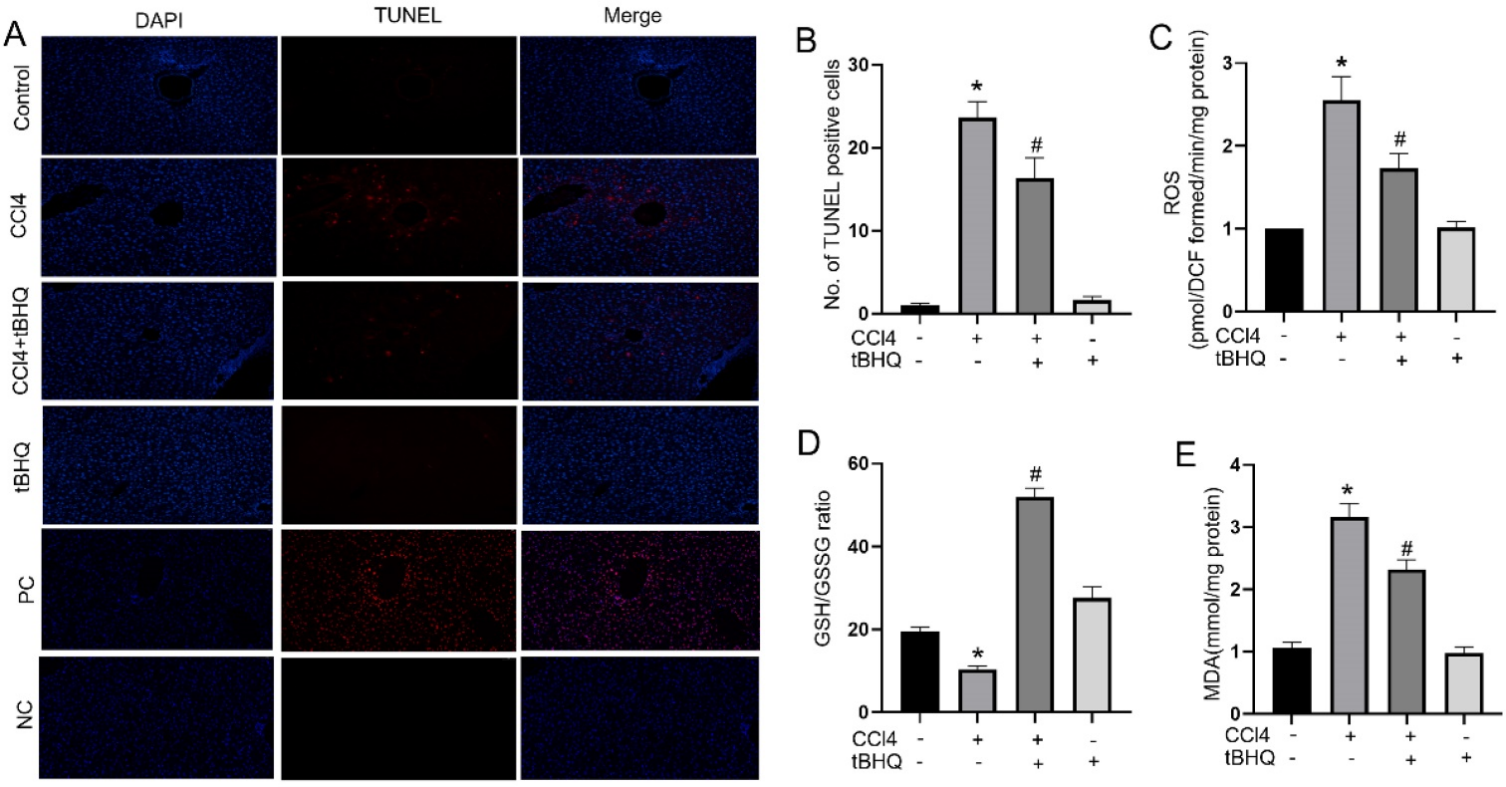
** Treatment of tBHQ reduced CCl4 induced apoptosis and oxidative stress in liver tissues.** TUNEL staining displayed that the TUNEL positive cells (red) in the CCL4 group were increased compared to control group in liver tissues (**A**). CCL4+tBHQ group significantly displayed fewer TUNEL positive cells compared to CCL4 group (A). Single tBHQ and control group had no TUNEL positive cells (A). A statistical analysis of TUNEL positive cells was performed (**B**). Levels of ROS (**C**), GSH/GSSG ratio (**D**) and MDA (**E**). PC means positive control group. NC means negative control group. Data were expressed as mean ± SEM (n = 6). *p < 0.05 vs. control; #*p* < 0.05 vs. the CCL4 group.

Oxidative stress and cell apoptosis are two mutual processes. Excessive oxidative stress can induce apoptosis in liver, which in turn can increase inflammation and producing ROS that further exacerbates cells death. In our study, we assessed whether tert-butylhydroquinone can protect hepatocytes from apoptosis. As shown in Fig. 2A, there were no TUNEL staining positive sites in control group. The TUNEL positive staining significantly increased after CCL4 treatment. However, after treatment with tert-butylhydroquinone TUNEL positive staining hepatocytes markedly decreased. In addition, treatment of tert-butylhydroquinone alone showed no impact on apoptosis of hepatocytes. Moreover, the positive control (PC) and negative control (NC) groups were showed in Fig. 2A. All cells were stained positive in PC group and no positive cells were showed in NC group.

Accumulation of ROS indicates oxidative stress which plays an important role in CCL4 induced hepatic injury. Therefore, we tested ROS generation via using DCFH-DA in each group of mice. As shown in Fig. 2C, ROS level increased significantly after CCL4 treatment compared to control group. In contrast, tert-butylhydroquinone decreased markedly generation of ROS in hepatic tissue compared to CCL4 group. To further investigate impact of tert-butylhydroquinone on CCL4 induced oxidative stress, we measured malondialdehyde levels and GSH/GSSG (oxidized GSH) ratio in each animal groups. Malondialdehyde is a pivotal parameter that can reflect organ lipid peroxidation rate and intensity of peroxidation damage. GSH/GSSG ratio is important mark to signify potential antioxidant capacity. In our study, we found that MDA content in CCL4 treated group was elevated significantly and administration of tert-butylhydroquinone can markedly decreased level of MDA (Fig. 2E). Besides, GSH/GSSG ratio was also increased significantly in CCL4+tBHQ group compared to CCL4 group (Fig. 2D).

### Tert-butylhydroquinone decreases CCL4 induced proinflammatory mediators

**Figure 3:**
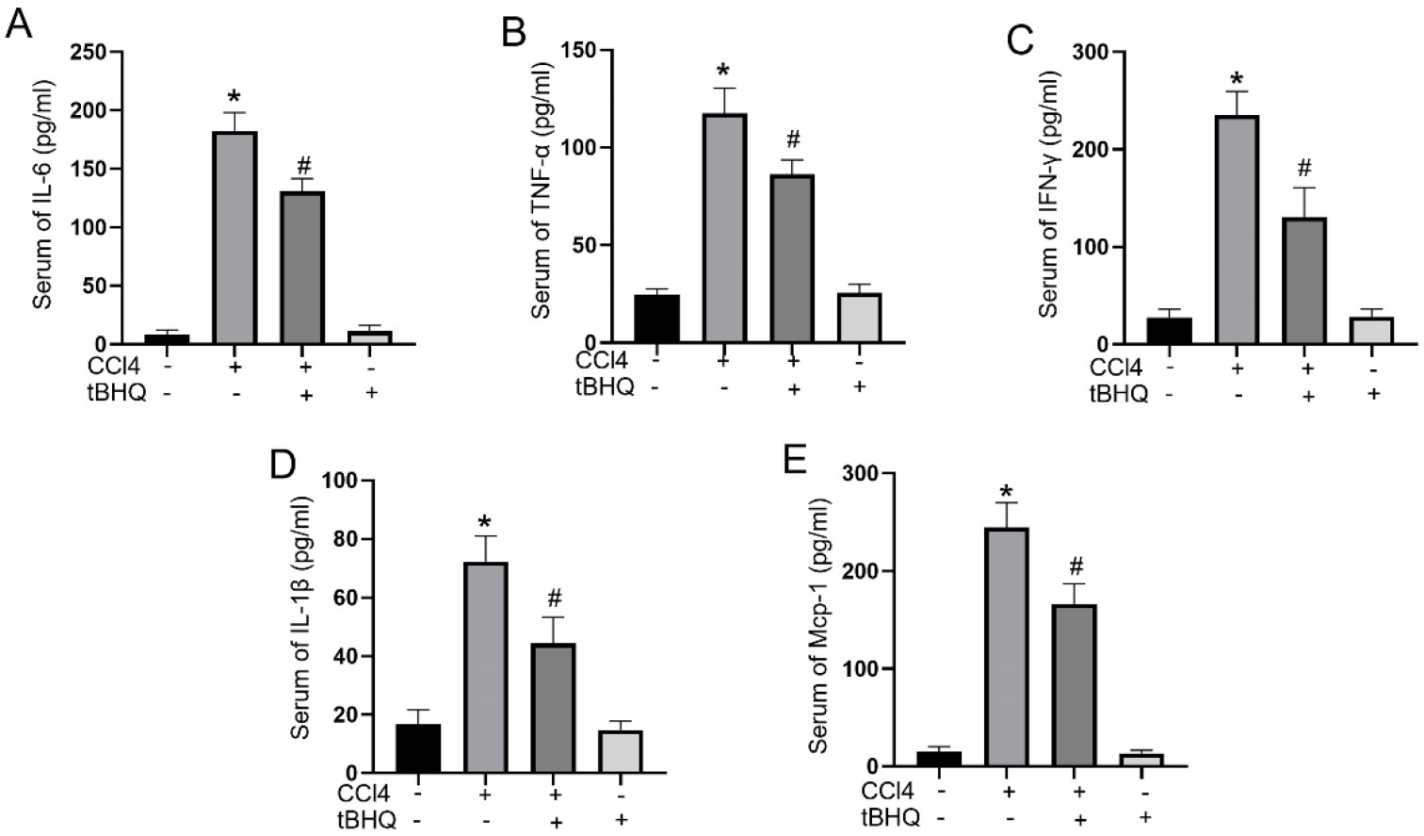
** Treatment of tBHQ inhibits proinflammatory cytokines in serum.** Activities of IL-6 (**A**), TNF-α (**B**), IFN-γ (**C**), IL-1β (**D**), and MCP-1 (**E**) in serum were analyzed by enzyme-linked immunosorbent assay in each group. Data were expressed as mean ± SEM (n = 6). *p < 0.05 vs. control; #*p* < 0.05 vs. the CCL4 group.

Cytokines play a pivotal role in acute liver injury. In mice experiments, administration of tert-butylhydroquinone alone showed no influences on activities of IL-6, IFN-γ, TNF-α, IL-1β, and MCP-1. CCL4 treatment can markedly elevated serum levels of proinflammatory mediators IL-6, IFN-γ, TNF-α, IL-1β, and MCP-1. Compared to CCL4 group, the activities of IL-6, IFN-γ, TNF-α, IL-1β, and MCP-1 in serum significantly decreased with tert-butylhydroquinone treatment (Fig. 3). This implied tert-butylhydroquinone inhibiting inflammatory response in CCL4 treated mice.

### Tert-butylhydroquinone reduces ROS production *in vitro*

**Figure 4:**
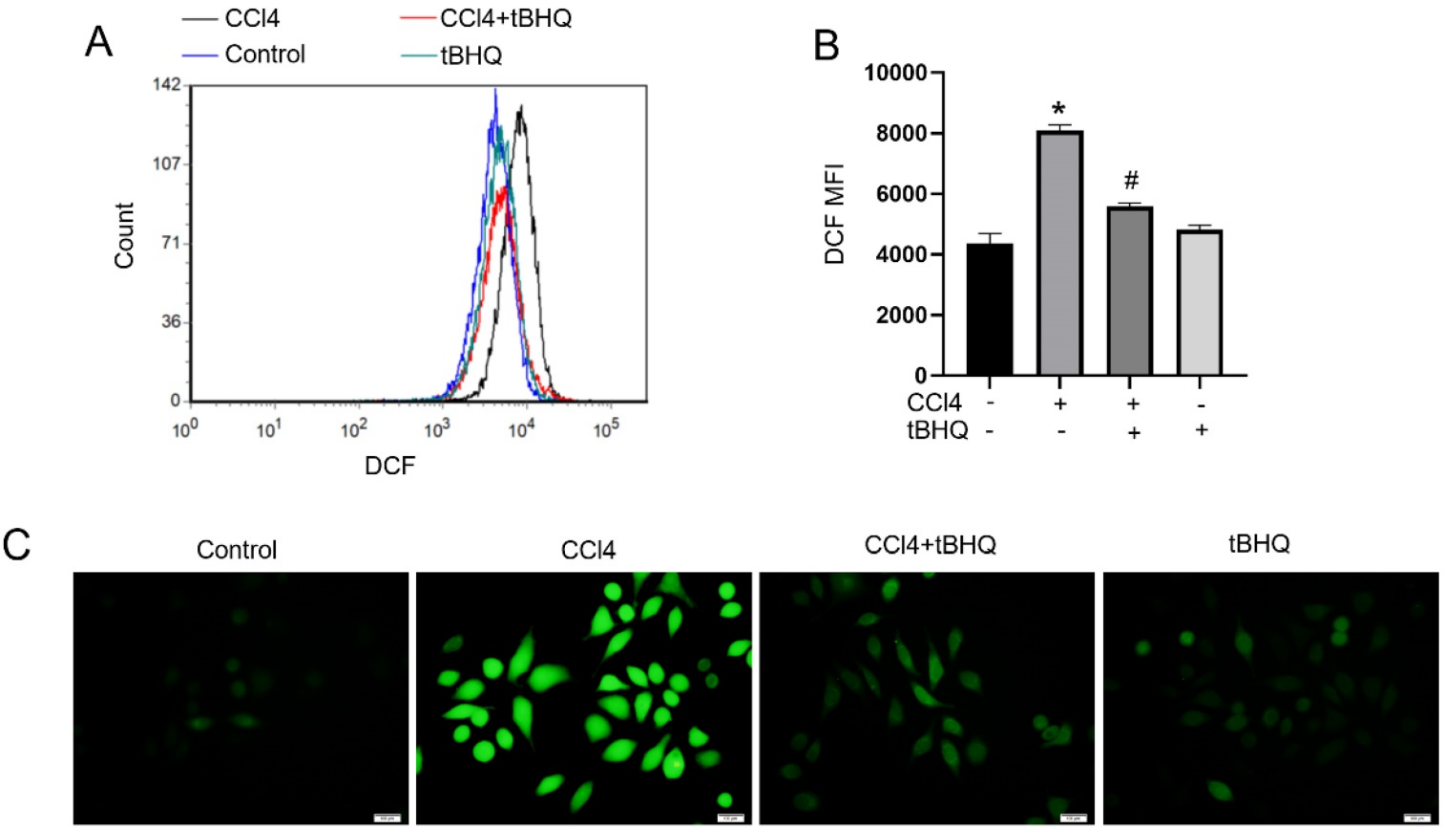
** Treatment of tBHQ decreases CCL4 induced production of ROS in cell line.** Histograms of DCF fluorescence (**A**). Mean fluorescence intensity (MFI) of each group (**B**). Images were captured after DCFH-DA probe staining using fluorescence microscope (**C**). All experiments were performed three times independently. Data were expressed as mean ± SEM. *p < 0.05 vs. control; #*p* < 0.05 vs. the CCL4 group.

To further explore function of tert-butylhydroquinone in hepatocytes. Hepatic carcinoma cell line HepG2 were used in our next experiments. We used DCFH-DA to determine ROS generation in HepG2 cells. Flow cytometric and fluorescence microscope observation found that CCL4 can induce ROS level markedly compared to control. And notably, after tert-butylhydroquinone treatment ROS level was significantly inhibited (Fig. 4A-C). Tert-butylhydroquinone alone showed no impact on ROS production.

### Tert-butylhydroquinone inhibits CCL4 induced apoptosis of HepG2 cells

**Figure 5:**
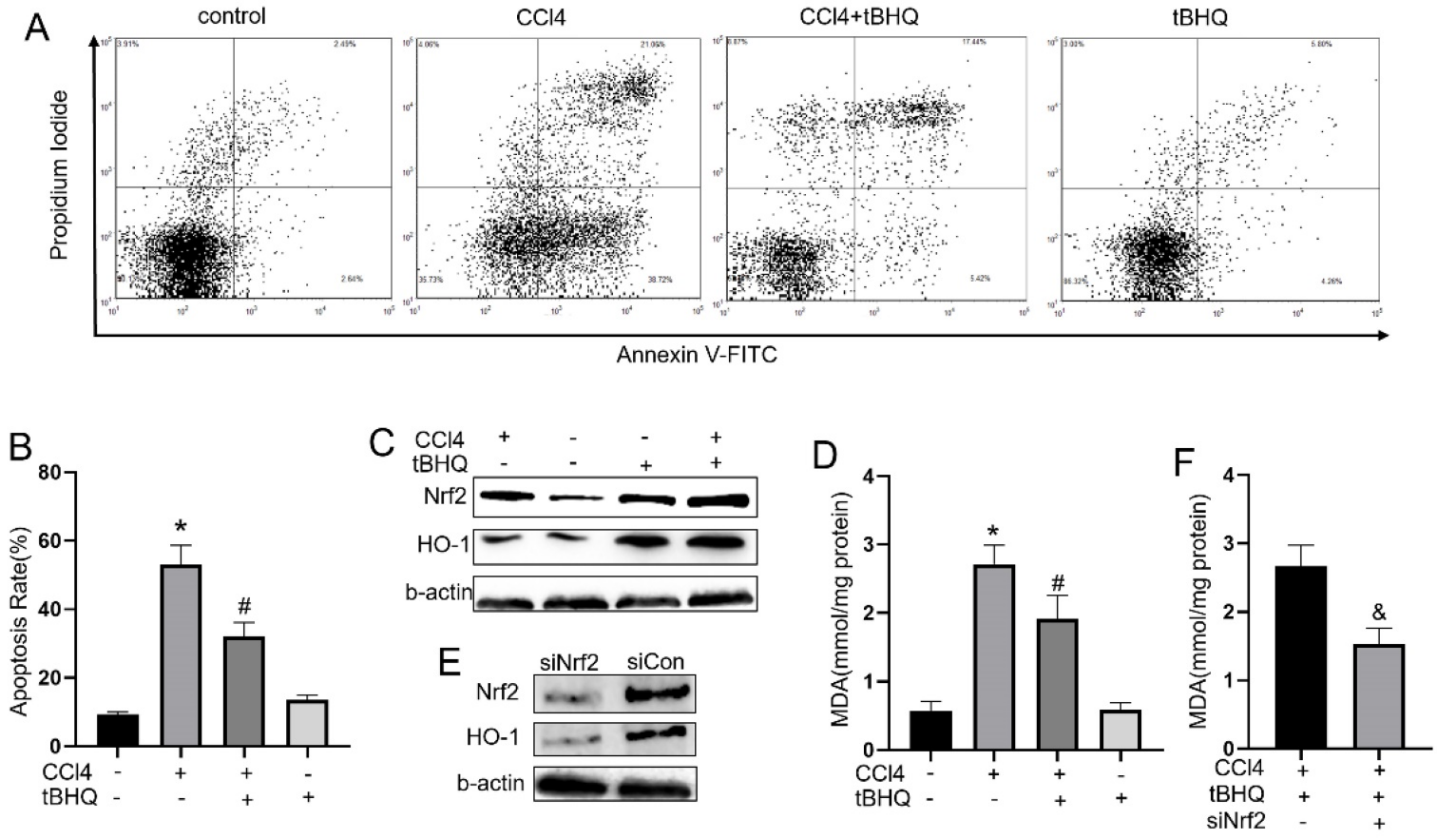
** Treatment of tBHQ inhibits HepG2 apoptosis and reduced oxidative stress by Nrf2/HO-1 pathway.** (**A**) The apoptosis of HeG2 cells was measured by flow cytometry and apoptosis rates was statistical recorded (**B**) of each group. (**C, G and H**) Expression of Nrf2 and HO-1 was evaluated with or without tBHQ and CCL4 treatment. levels of MDA in HepG2 cells were analyzed in each group (**D**). (**E, I and J**) Nrf2 was knock down with siRNA-Nrf2 (siNrf2), Nrf2 and HO-1 levels reduced compared to siRNA-control (siCon) with existence of CCL4 and tBHQ in HepG2 cells. (**F**) Knock down Nrf2 increased MDA level in HepG2 cells treated with CCL4 and tBHQ. All experiments were performed three times independently. Data were expressed as mean ± SEM. *p < 0.05 vs. control; #*p* < 0.05 vs. the CCL4 group; &p < 0.05 vs. the siCon group.

To explore whether tert-butylhydroquinone can exhibit protective effect in CCL4 treated hepatocytes *in vitro*, we measured apoptosis rate in CCL4 treated HepG2 cells with or without tert-butylhydroquinone using flow cytometry. As shown in fig.5A and 5B, CCL4 significantly induced cells apoptosis compared control group. However, treatment of tert-butylhydroquinone reduced apoptosis rate on CCL4 treated HepG2 cells. In addition, single tert-butylhydroquinone treatment had no impact on apoptosis of HepG2.

### Tert-butylhydroquinone protects hepatocytes via activating Nrf2 pathway

To explore underlying mechanism of tert-butylhydroquinone protective effect, we measured Nrf2, and HO-1 expression in HepG2 cells. As shown in fig.5C, 5G and 5H, Nrf2 and HO-1 expressed at a low level without CCL4 and tert-butylhydroquinone. Treatment cells with CCL4 without tert-butylhydroquinone can elevate Nrf2 while HO-1 expressed at low level. Single tert-butylhydroquinone treatment can significantly increase expression of Nrf2 and HO-1. Treatment cells with CCL4 and tert-butylhydroquinone showed the highest expression of Nrf2 and HO-1. In addition, we measured MDA level in cells with or without tert-butylhydroquinone and CCL4 treatment. We found CCL4 increased MDA while addition of tert-butylhydroquinone significantly reduced MDA level (Fig. 5D).

Then cells were transfected Nrf2-siRNA to knock down expression of Nrf2. Our data exhibited siNrf2 group showed markedly lower expression of Nrf2 and HO-1 compared to siCon group under CCL4 and tert-butylhydroquinone treatment (Fig. 5E, 5I and 5J). Moreover, knock down Nrf2 markedly increased MDA level in CCL4 and tert-butylhydroquinone treated HepG2 cells (Fig. 5F).

## Discussion

Many toxic compounds can disturb hepatic physiological function and metabolism. Oxidative stress is one of most important factors inducing liver damage 13. CCl4 is a chemical can induce oxidative stress and cause damage in hepatocytes. TBHQ is an anti-oxidants compound widely used in food industry. In addition, it is an agonist of Nrf2 14. Activators and inhibitors of Nrf2 are potential in clinical use 15. Nrf2 agonist, such as dimethyl fumarate, was confirmed effective in clinical treatment of multiple sclerosis and reducing relapse rate after remission in multiple sclerosis patients 16. Given that Nrf2 pathway is crucial in regulating oxidative stress and inflammatory response, tBHQ is a promising potential drug that could be effective in treatment certain diseases. And experiments in animals validated its efficiency. *In vivo*, treatment of tBHQ showed markedly cytoprotective effect in many tissues under pathological condition 17, 18. Administration of tBHQ could inhibit ROS generation and reduce renal injury in renal ischemia and reperfusion model 19. Continuous tBHQ treatment markedly decreased and ameliorated proteinuria in type 1 diabetes mice 20. Also, intra-cerebroventricular administration with tBHQ significantly reduced size of infraction and neurological disfunction was improved 21, 22. Moreover, tBHQ protected hepatocytes from saturated fatty acids induced cells death by enhancing autophagy 14. However, the impact of tBHQ in CCL4 induced ALI has not previously been investigated.

In our present study, we found treatment of CCL4 increased liver injury as we described before 12. Serum ALT and AST levels are widely used to evaluate hepatic function. We found tBHQ can alleviate CCL4-induced elevation of AST and ALT. Furthermore, tBHQ decreased histologic hepatic damage and inhibited apoptosis of hepatocytes. CCL4 is metabolized by liver CYPs to CCL 3• free radicals and lead to lipid peroxidation and oxidative stress. In normal condition, ROS is generated and eliminated by antioxidant defense system 23. Cells can response to oxidative stress when exposed to ROS and electrophilic chemicals by expression of protective proteins to avoid harm to cells. Nevertheless, when cells encountered excessive ROS that exceed capacity of defense system, it leads to structural damage and dysfunction and consequently results in necrosis, apoptosis, inflammation and other disorders 24, 25. Therefore, eliminating ROS is pivotal in protecting organ or function of cells. Under treatment of tBHQ, the levels of ROS in liver tissues were significantly decreased. GSH/GSSG is one of the most important redox pairs that can reflect status of oxidative stress. MDA is final product of lipid peroxidation. Correspondingly, GSH/GSSG ratio and MDA level reduced under tBHQ administration, implying anti-oxidative ability of tBHQ. Moreover, we analyzed ROS generation in CCL4 treated cells *in vitro* via DCFH-DA. The ROS level in CCL4 treated cells increased markedly while treatment of tBHQ could significantly reduce level of ROS. All these results indicated that tBHQ can protect cells from CCL4 induced oxidative stress damage *in vivo* and *in vitro*.

Excessive inflammation is another major cause implicated in CCL4 induced hepatic damage 26. Proinflammatory cytokines such as IL-1β, IL-6, INF-γ, TNF-α and MCP-1 are important in pathological process of ALI. Anti-TNF-α antibody could mitigate CCL4 induced hepatic injury in a previous report 27. Also, IL-1β was reported played pivotal role in maintenance inflammatory condition 28. Expression of IL-6, IFN-γ, and MCP-1 were significantly elevated in ALI tissues and led to development of inflammation 29. In addition, excessive free radicals can activate hepatic macrophages to intense proinflammatory reaction via producing proinflammatory cytokines 30. In our study, treatment of tBHQ significantly reduced levels of proinflammatory cytokines in CCL4 treated mice, which is crucial in mitigating liver injury.

We further explored the possible mechanism of this protective effect *in vitro*. Nrf2 is a pivotal factor in oxidative stress response. Increasing evidence showed that Nrf2 displayed cytoprotective effects when cells were exposed adverse condition 31-34. Nrf2 regulates ranges of antioxidants gene expression via binding to antioxidant response element. HO-1 is directly regulated by Nrf2 under oxidative stress 6. HO-1, as a stress-response enzyme, can degrade heme to carbon monoxide, ferrous iron and biliverdin. The degradation of heme group is beneficial to prevent its oxidation promoting effect. Moreover, by-product biliverdin and its reduced bilirubin have an effective ROS scavenging activity to resist peroxide, peroxynitrite, hydroxyl and superoxide radicals. Nrf2/HO-1 pathway exhibited potential clinical value in some diseases. Activation of Nrf2/HO-1 showed protective effects in cardiovascular system and exhibits function in anti-inflammation, anti-apoptosis and anti-oxidative processes 35. Moreover, activation of Nrf2/HO-1 could significantly mitigate neurotoxicity via reducing oxidative stress and inflammation 36. In our study, we confirmed tBHQ could activate Nrf2 and increase HO-1 expression. Treatment with tBHQ induced more Nrf2 and consequent HO-1 expression compared in CCL4 treated HepG2 cells. Cells showed less level of MDA under activation of Nrf2/HO-1, which signified Nrf2/HO-1 pathway was pivotal in resisting oxidative injury caused by CCL4. Then we designed siRNA of Nrf2 to inhibit Nrf2 expression in HepG2 cells and treated cells with tBHQ and CCL4 together. After expression of Nrf2/HO-1 was inhibited, we found treatment of tBHQ showed increasing production of MDA with CCL4 administration. Therefore, activation Nrf2/HO-1 pathway is associated with protective effects of tBHQ in CCL4 induced liver damage.

## Conclusion

In conclusion, our present study suggests CCL4 promote liver injury. Treatment of tBHQ attenuates liver injury by inhibiting oxidative stress, inflammation and cells apoptosis. The possible mechanism of protective effect of tBHQ may be associated with activation of Nrf2/HO-1 pathway. Taken together, our data suggests that tBHQ could be a potential therapeutic drug in treatment of acute liver injury.

## Abbreviations

ALI: acute liver injury
tBHQ: Tert-butylhydroquinone
CCL4: carbon tetrachloride
ROS: reactive oxygen species
ALT: transaminase
AST: aspartate transaminase
MDA: malondialdehyde
ELISA: enzyme-linked immunosorbent assay
IL-6: interleukin-6
IL-1β: interleukin-1β
IFN-γ: interferon-γ
MCP-1: monocyte chemotactic protein 1
TNF-α: Tumor necrosis factor-α
DCFH-DA: 2,7-Dichlorofluorescein diacetate
MFI: Mean fluorescence intensity
NRF2: Nuclear factor E2-related factor 2
TUNEL: terminal dexynucleotidyl transferase(TdT)-mediated dUTP nick end labeling
